# Prevalence of Subclinical Mastitis and Distribution of Pathogens in Dairy Farms of Rubavu and Nyabihu Districts, Rwanda

**DOI:** 10.1155/2017/8456713

**Published:** 2017-07-17

**Authors:** J. P. Mpatswenumugabo, L. C. Bebora, G. C. Gitao, V. A. Mobegi, B. Iraguha, O. Kamana, B. Shumbusho

**Affiliations:** ^1^Department of Veterinary Medicine, University of Rwanda, P.O. Box 210, Musanze, Rwanda; ^2^Department of Veterinary Pathology, Microbiology and Parasitology, University of Nairobi, P.O. Box 29053-00625, Kangemi, Kenya; ^3^Department of Biochemistry, University of Nairobi, P.O. Box 30197, Nairobi, Kenya; ^4^Rwanda Dairy Competitiveness Program II, P.O. Box 569, Kigali, Rwanda; ^5^Department of Food Safety and Food Quality Management, University of Rwanda, P.O. Box 210, Musanze, Rwanda

## Abstract

A cross-sectional study was conducted from May 2016 to January 2017 in Rubavu and Nyabihu districts, Western Rwanda, aiming at estimating the prevalence of subclinical mastitis (SCM) and identifying its causative bacteria. Management practices and milking procedures were recorded through a questionnaire. 123 crossbreed milking cows from 13 dairy farms were randomly selected and screened for SCM using California Mastitis Test (CMT). Composite CMT positive milk samples were processed for bacterial isolation and identification. The overall SCM prevalence at cow level was 50.4%. 68 bacterial isolates were identified by morphological and biochemical characteristics. They included, Coagulase Negative Staphylococci (51.5%),* Staphylococcus aureus* (20.6%),* Streptococcus* species (10.3%),* Bacillus* species (10.3%),* Streptococcus agalactiae* (5.8%), and* Escherichia coli* (1.5%). About 67.1% of the farmers checked for mastitis; of these, 58.9% relied on clinical signs and only 6.8% screened with CMT. Only 5.5% and 2.7% of the farmers tried to control mastitis using dry cow therapy and teat dips, respectively. Thus, to reduce the prevalence of SCM, farmers in the study area need to be trained on good milking practices, including regular use of teat dips, application of dry cow therapy, and SCM screening. This will improve their sales and their financial status.

## 1. Introduction

Mastitis is defined as inflammation of mammary gland. It is divided into two types: clinical and subclinical. Clinical mastitis (CM) is characterized by visible changes in milk (e.g., clots, color changes or consistence, and decreased production) that may be associated with inflammation signs of the udder (e.g., redness, swelling, heat, or pain) or the cow (e.g., dehydration, hyperthermia, and lethargy) [1]. SCM is asymptomatic; therefore, produced milk appears to be normal. On the other hand, according to the course of the disease and the severity of the inflammatory response, mastitis may be classified as peracute, acute, subacute, and chronic [2].

According to [3], mastitis is the major disease that affects the dairy subsector. Different studies have shown mastitis to be one of the most costly diseases of the dairy industry worldwide [4, 5]. Several economic losses result due to mastitis such as reduction of milk yields, milk discards due to bacterial or antibiotic contamination, veterinary intervention costs, and occasionally deaths [6].

While it is easy to detect CM (seeing clotted milk), SCM can only be demonstrated using various tests such as California Mastitis Test (CMT), Whiteside test (WST), Surf field mastitis test (SFMT), sodium lauryl sulphate test (SLST), Microscopic Somatic Cell Count (MSCC) [7, 8], and Electrical Conductivity (EC) [9]. Enzymatic analyses such as colourimetric and fluorometric assays have also been developed [10]. New advanced techniques such proteomics have been recently developed and used in detection of proteins involved in mastitis [11–13]. Most of these tests are preferred as screening tests indicating SCM since they are easy to use and yield rapid as well as satisfactory results. However, CMT has been recognized as a highly sensitive test to detect bovine subclinical mastitis [14, 15]. It has been reported by [8] that the sensitivity of the CMT was 86.1 while specificity was 59.7% with percentage accuracy of 75.5%. In a similar study, [14] found that sensitivity for the Modified California Mastitis Test (MCMT) was 95.2% while its specificity was 98.0%. In order to identify mastitis causing microorganisms, the microbiological culture procedures still are the gold standard [10].

Different studies have shown that SCM is mainly caused by Coagulase Negative Staphylococci (CNS),* Staphylococcus aureus (S. aureus)*,* Streptococcus agalactiae (Str. agalactiae*), other* Streptococcus* species, and coliforms [9, 16].

So far, the prevalence of SCM and causative bacteria in lactating cows of Rubavu and Nyabihu district in Rwanda is not known. Therefore, this study was conducted to determine the prevalence of subclinical mastitis as well as isolate and identify the bacterial agents associated with SCM in lactating cows in these two districts and to assess possible association with SCM within the two production systems (extensive and intensive). Milking procedures and management practices that influence the prevalence of mastitis in the study area were also evaluated.

## 2. Materials and Methods

### 2.1. Study Area

The current study was carried out in Rubavu and Nyabihu districts which are located in the Western Province of Rwanda (−1°40′52.54′′S, 29°19′45.55′′E and −1°39′9.90′′S, 29°30′24.62′′E, resp.). They are 152 km far from Kigali, the capital city of Rwanda, and are boarded by Democratic Republic of Congo (DRC) through Virunga National Park (VNP) and Lake Kivu. The altitude varies from 1830 to 2437 m in Rubavu and Nyabihu, respectively.

Rubavu district has an average annual temperature of 18.1°C with an average annual rainfall of 1,377 mm whereas Nyabihu district has average annual temperature of 15°C and rainfall reaching 1,400 mm per year. Their main types of soils can be grouped into three categories: volcanic soils, lateritic and humus-bearing soils, and clayey soils.

Rubavu and Nyabihu districts are characterised by two dry seasons as well as two rainy seasons: the long dry one stretches from June to mid-September and the short one from January to mid-March; the long rainy season stretches from March till the end of May and the short rainy season from September to December. Heaviest rains fall in April and May, whereas moderate rains fall in October and November.

### 2.2. Sample Size and Sampling Method

The study was carried out on 123 lactating crossbreed (Friesian versus Ankole and Jersey versus Ankole) cows, 61 from intensive system and 62 from extensive system randomly selected from 13 smallholder dairy farmers, 6 in Rubavu and 7 in Nyabihu. The sample size was determined by using the formula stated by [17]. The basis for sampling was the production system (extensive or intensive) practiced by dairy farmers in the study area. Extensive system was defined as production system where livestock are left to wander and graze during the day and are enclosed during the night whereas intensive system was defined as a production system where cows are kept in zero grazing, being served with grass, supplements, and water, and spend the night in kraal. During farm visits, a structured questionnaire was used to collect information at herd and animal levels regarding herd size, milk production, record keeping, milking practices, mastitis screening, and control measures. Observational assessment was also made on the hygiene of animals as well as cow sheds.

### 2.3. California Mastitis Test and Detection of SCM

Prior to milk collection for mastitis screening, clinical examination was performed on the every lactating cow. Thorough palpation of the udder to detect any fibrosis, swelling, and other clinical signs was performed. Watery milk, milk with pus or clots, and blind quarters were also examined. Identification of at least one of these signs was enough to consider the mammary quarter as positive to CM and was excluded from the study [16].

Subclinical mastitis prevalence was obtained by the use of California Mastitis Test (CMT) which was conducted using scores from 0 to 4 from the modified Scandinavian scoring system, where 0 is negative result (no gel formation), 1 is traceable (possible infection), and 2 or 3 indicates a positive result and 4 has the thickest gel formation. A sample was defined as positive to SCM when one or more quarters with CMT ≥ 2+ were detected [18].

Milk samples were collected from all four quarters and individually analysed with CMT to detect SCM, as previously described [16]. After confirming SCM by CMT, the udder and teats were cleaned with water and wiped using sterile towels. The teat orifice and the skin around the teat were sprayed with 70% alcohol and dried off with sterile towels.

### 2.4. Processing of Milk Samples and Bacteriological Assays

The samples were taken shortly prior to milking and only cows expressing no clinical signs of mastitis were sampled. Composite milk samples from CMT positive cows (all cows whose composite milk tested positive to CMT) were aseptically collected directly from quarters into aseptic tubes and taken to the laboratory for bacteriological analysis to identify SCM causative microorganisms [19].

Milk samples were bacteriologically examined according to the procedure previously described [20]. After reaching the laboratory (1-2 hours), milk samples were aseptically removed from the cooler box for examination. Composite CMT positive milk samples were inoculated separately onto MacConkey agar and Blood agar plates by streaking method. Inoculated plates were then incubated aerobically at 37°C for 24–48 hours. After 24 hours, primary bacteriological identification was made based on colony morphology, color, and haemolytic characteristics; these were considered as pure or individual cultures. After primary culture readings, pure cultures were prepared through subculturing and incubation. The purified isolates were then subjected to Gram staining and further biochemical testing. Staphylococci were identified based on catalase test and tube coagulase test. Streptococci were identified based on catalase and Christie, Atkins, and Munch-Peterson (CAMP) test. Gram negative isolates were identified based on growth characteristics on MacConkey agar and reactions to strip oxidase test, catalase test, Triple Sugar Iron (TSI) agar, and the “IMViC” tests (Indole, Methyl-Red, Vogas Proskaur and Citrate utilisation) [16]. Composite CMT results, culture, Gram stain, and readings of biochemical tests were encoded in excel spreadsheet to determine the prevalence of SCM and related causative bacteria. Milk samples were collected during the rainy season.

### 2.5. Descriptive Statistics

Information regarding respondents' particulars (age, sex, and level of education), herd characteristics, management practices, and milking procedures were encoded into excel spreadsheet for descriptive analysis. Correlations between production systems and prevalence of SCM were computed by Statistical Package for Social Sciences (SPSS).

## 3. Results

Respondent's identification and herds characteristics were presented (Table 1). 100% of the studied animals were crossbreed of Friesian and Ankole (local cattle) while milk production per day doubled in intensive system compared to extensive system. On the other hand, following our field observations, it was noticed that milking practices and procedures are inadequate in the study area (Table 2). Management practices employed by farmers in the study area were reported (Table 2). Among 67.1% of dairy farmers who screen for mastitis only 6.8% use CMT while 58.9% observe appearance of clinical signs. Out of 84.9% who control mastitis and 2.7% use teat dips, 65.8% treat clinical mastitis cases while 5.5% apply dry cow therapy. 100% of all farmers in the study area milk their cows by hands (Table 2) while 89% of dairy farmers milk their cows in open space.

The overall SCM prevalence at cow level was 50.4% (62/123) (Table 3), prevalence being higher in Rubavu district (intensive system) 61.3% (38/61) than in Nyabihu district (extensive system) 38.7% (24/62). However, the differences between these two farming systems were not statistically significant (*P* = 0.087, CI = 95%) (Table 3).

From a total of 62 composite SCM positive samples cultured, 68 bacterial isolates were identified (Table 4); 6 samples contained more than one organism which were* S. aureus* and CNS while the other 56 samples were associated with single infection. In this study, the most predominant bacteria were CNS at 51.5% (35/68) followed by* S. aureus* at 20.6% (14/68), other* Streptococcus* species at 10.3% (7/68), Bacillus spp. at 10.3% (7/68), and* Str. agalactiae* at 5.8% (4/68) and the least was* Escherichia coli* (*E. coli*) at 1.5% (1/68) (Table 4).

## 4. Discussion

The results from this study show a high prevalence of SCM. A possible explanation for this finding could be that most farmers in the study area do not practice proper farming management and screen for mastitis at earlier stage. Results from the survey have revealed that only 6.8% screen for SCM using CMT, 58.9% only observe appearance of clinical signs which is difficult in SCM, and 32.9% do not screen for mastitis. This could also be supported by the fact that 97.3% of farmers in the study area do not practice teat dipping.

The current findings corroborate with those reported in recent studies and in the same country (51.8%) [21] using electrical conductivity and in Tanzania (51.6%) [22, 23], in Ethiopia, all have used CMT to screen for SCM at cow level. It was also similar to those reported from other countries: 49.5%, 51.8%, and 52.4% in South Wales in Australia [24], in Bangladesh [25], and in Uruguay [26], respectively, all using CMT. However, this reported that SCM prevalence was lower than those reported in recent studies in East Africa; 86.2%, 64% and 59.2% in Uganda [27], in Kenya [28], and [23] in Ethiopia, respectively, and elsewhere, 88.6%, by [29] in Vietnam all have used CMT to screen for SCM at cow level. These differences should be due to different screening methods used. For instance, [29] used SCC determinations with or without positive isolation of pathogens to determine SCM while CMT was used in the current study. It has also been noted that the high prevalence recorded in Kenya [28] was attributed to the breed; 62.8% of the studied animals were Friesian and Jersey. However, in the current study, all cows were crossbreeds which are less prone to mastitis than exotic breeds [30]. In contrast, the prevalence reported in the current study was higher than 34.1% reported by [31] in Njoro District of Kenya, 41.0% by [32] in Ethiopia, 42.5% by [33] in Iran, 28.5% by [34] in Bangladesh, all of which used CMT test. These difference should be supported by farming systems and management practices and cow breeds. The authors of [31] in Kenya has screened for SCM on cows reared in paddocks where animals are grazed on the green pasture. This is also supported by [35] in UK who found that grass-based herds were less exposed to environmental bacteria, hence less prevalence of subclinical mastitis. On the other hand, the authors of [34], using CMT, have found a low SCM prevalence because 74% of their study animals were local breeds (zebu) which are less prone to mastitis [30].

According to [36], mastitis prevalence of 40 % or higher in a farm must sound alarming to the producer; hence, this study reveals how serious mastitis is the problem in the dairy industry sector of Rwanda; it requires attention. Pre- and postmilking teat disinfection have been recommended as important procedures to prevent prevalence and incidence of mastitis [37]; however, it is not practised in any of the farms in the current study.

The distribution of CNS as the most predominant bacteria isolated from the CMT positive samples, followed by* S. aureus* and* Streptococcus* species in this study, is confirmed by [38]. In a similar way, [39] found the CNS, coagulase positive staphylococci (CPS;* S. aureus*), the environmental streptococci, and coliforms as the prevalent mastitis pathogens associated with SCM in lactating cows. The predominance of CNS in SCM in this study is also in line with the findings of [40] in Czech Republic, [27, 41] in Uganda, and [42] in Canada. The high predominance of CNS in the current study areas can be explained by poor milking hygienic practices in the farms, coupled by nonuse of teat dips and lack of routine mastitis screening tests; these provide an opportunity for the CNS to invade the udder and develop into an intramammary infection. It is also stated that staphylococcal mastitis is the most common form of contagious mastitis and these organisms are spread from infected to clean cows on hands or equipment from one udder to another [43].

CNS are considered to be teat skin opportunists that normally reside on the teat skin and cause mastitis via ascending infection through the teat canal [44]. However, recent reports suggest that CNS have become the most common bovine mastitis isolates in many countries and could therefore be described as emerging mastitis pathogens [45–47].

The prevalence rate of* S. aureus* (20.6%) in the current study agrees with previous findings by authors of [30, 38, 39] who reported* S. aureus* to be the most predominant bacterial isolate in their studies. Being a contagious pathogen [48],* S. aureus* prevalence rate could be associated with poor milking hygiene and lack of teat dipping in the current study. It has been reported that* S. aureus* has adaptive mechanisms that allow it to be shed on the udder and cause intramammary infections during milking processes [49]. In some studies,* S. aureus* are the second most prevalent pathogens, while in other studies the environmental mastitis pathogens are more prevalent [1]. As reported by [21] in Eastern Rwanda, coliform bacteria were mostly isolated from SCM positive milk samples. It should, however, be noted that Iraguha's study was carried out during dry season (where there was contamination by soil and fecal matter) whereas the current study was conducted during the short rainy season.

Although environmental streptococci (10.3%) were ranked third followed by* Str. agalactiae* (5.8%) in the current study, [29] in Vietnam reported* S. agalactiae* as the most predominantly (21%) isolated bacteria. Similar findings have been reported by [9] who found that* Streptococci* spp. ranked the second among all isolates from subclinical mastitis with a rate of 26.3%. These findings are also in line with these reported by [50] in Uganda, who found* S. agalactiae* at 8.4% in SCM.* S. agalactiae* has been associated with SCM and it can also cause CM [9].

Although* Bacillus* spp. have been reported to be an uncommon cause of mastitis in cattle [51] and affected animals express acute to gangrenous form of mastitis [52], this species has been reported in the current study at a slightly high rate. This could be explained by the poor hygienic conditions of milkers in the study area. It has been found that* Bacillus* spp. are widely distributed in nature and most species exist in soil, in water, in dust, in air, in feces, and on vegetation [53]. Therefore,* Bacillus* spp. should be considered as a cause of intramammary infection in a cow with high SCC or clinical signs of udder disease; otherwise, the presence of few* Bacillus* spp. colonies on blood agar would be expressed as contamination [53].

The findings of the current study have shown a low prevalence of* E. coli*, though the study was conducted during the rainy season, case which should depict the opposite findings as coliforms being environmental bacteria associated with wet and muddy conditions [54]. However, the same authors have reported coliform bacteria to be of more importance in CM than subclinical mastitis, despite the environmental factors. This confirms that the prevalence of coliform bacteria in this study would be low, as the selection criteria identified cows with SCM as opposed to CM. On the other hand, coliforms have been confirmed to commonly be involved in CM characterized by a rapid onset associated with acute and peracute forms [16], of short duration [55], and rarely cause SCM [50]. These findings corroborate with those reported in Tanzania, whereby [56] found that SCM was associated with coliforms at 4.1%. However, it has been found that chronic and subclinical infections occur and recurring infections with* E. coli* may be more common than previously thought [57] and could also be associated with immune-depressed animals [58].

The poor management and udder health practices, inadequate milking procedures observed by the farmers and milkers, would expose the cows to SCM caused by environmental and contagious bacteria during milking by miller's hands, as was mostly found in this study. On the other hand, nonuse of teat dips and other mastitis control techniques due to lack of knowledge should have greatly contributed to the high prevalence of SCM in the study area. Farmers in the study area, therefore, need to be educated and encouraged to practice good farming, animal health management practices, and milking practices at all times; this will reduce udder contamination and subsequent SCM or CM. Adequate proper housing with sanitation, regular screening for early detection, and appropriate treatment of subclinical cases, dry cow therapy, and application of pre- and postdipping practices are also highly recommended.

## Figures and Tables

**Table 1:** Characterization of respondents and herds per production system in the study area.

Parameter	Intensive (*n* = 10)		Extensive (*n* = 63)		Total percentage
	Number of responses	Percentage	Number of responses	Percentage	
Sex					
Male	10	13.7%	60	82.2%	**95.9%**
Female	0	0.0%	3	4.1%	**4.1%**
Age					
[21–30]	0	0.0%	1	1.4%	**1.4%**
[31–40]	3	4.1%	6	8.2%	**12.3%**
[41–50]	2	2.7%	23	31.5%	**34.2%**
>50	5	6.8%	33	45.2%	**52.1%**
Education level					
Informal	1	1.4%	10	13.7%	**15.1%**
Primary	5	6.8%	46	63.0%	**69.9%**
Secondary	1	1.4%	4	5.5%	**6.8%**
University	3	4.1%	3	4.1%	**8.2%**
Cattle breed					
Cross breeds	10	13.7%	63	86.3%	**100.0%**
Herd size (mean)	30	—	21	—	**—**
Lactating cows (mean)	14	—	10	—	**—**
Milk production (mean L/day)	110	—	53	—	**—**
Milking frequency/day					
Once	0	0.0%	0	0.0%	**0.0%**
Twice	10	13.7%	63	86.3%	**100.0%**

**Table 2:** Management practices routines employed by dairy farmers in the study area.

Management practice	Intensive (*n* = 10)		Extensive (*n* = 63)		Total percentage
	Number of responses	Percentage	Number of responses	Percentage	
Mastitis screening					
Yes	6	8.2%	43	58.9%	67.1%
No	4	5.5%	20	27.4%	32.9%
If yes, how?					
CMT	3	4.1%	2	2.7%	6.8%
Strip cup	0	0.0%	1	1.4%	1.4%
Clinical signs	3	4.1%	40	54.8%	58.9%
If no, why?					
Lack of knowledge	3	4.1%	17	23.3%	27.4%
Lack of screening materials	1	1.4%	1	1.4%	2.7%
No mastitis cases	0	0.0%	2	2.7%	2.7%

Mastitis control					
Yes	10	13.7%	52	71.2%	84.9%
No	0	0.0%	11	15.1%	15.1%
If yes, how?					
Cow hygiene	1	1.4%	7	9.6%	11.0%
Dry cow therapy	0	0.0%	4	5.5%	5.5%
Use of teat dips	2	2.7%	0	0.0%	2.7%
Treatment of clinical cases	7	9.6%	41	56.2%	65.8%
If no, why?					
Lack of knowledge	0	0.0%	11	15.1%	15.1%

Milking technique					
Hand milking	10	13.7%	63	86.3%	100.0%
Milking machine	0	0.0%	0	0.0%	0.0%

Milking place					
Open space	2	2.7%	63	86.3%	89.0%
Milking from stanchion/tie stalls	8	11.0%	0	0.0%	11.0%

**Table 3:** Subclinical mastitis prevalence in relation to production system.

Area (district)	Production system	Number of tested cows	Number of CMT mastitis positive	Number of CMT mastitis negative	% mastitis positive	*P* *value*
Rubavu	Intensive	61	38	23	61.3	0.087
Nyabihu	Extensive	62	24	38	38.7	

Total		**123**	**62**	**61**		

Overall prevalence					**50.4%**	

**Table 4:** Prevalence of bacterial agents isolated from CMT subclinical mastitis positive.

Number of samples for bacteriological culture	Bacterial isolates	Number of isolates	Prevalence
62	*S. aureus*	14	20.6%
	CNS	35	51.5%
	*Bacillus *spp.	7	10.3%
	*Str. agalactiae*	4	5.8%
	Other streptococci	7	10.3%
	*E. coli*	1	1.5%

## References

[1] Fox L. K. (2009). Prevalence, incidence and risk factors of heifer mastitis. *Veterinary Microbiology*.

[2] Mastitis Clinical Syndromes, http://ansci.illinois.edu/static/ansc438/Mastitis/syndromes.html

[3] Chatikobo P. (2010). Mastitis control for quality milk. *Dairy Mail Africa*.

[4] Biffa D., Debela E., Beyene F. (2005). Prevalence and risk factors of mastitis in lactating dairy cows in Southern Ethiopia. *International Journal of Applied Research and Veterinary Medicine*.

[5] Kossaibati M. A., Esslemont R. J. (1997). The costs of production diseases in dairy herds in England. *The Veterinary Journal*.

[6] Vaarst M., Enevoldsen C. (1997). Patterns of clinical mastitis manifestations in Danish organic dairy herds. *Journal of Dairy Research*.

[7] Hoque M. N., Das Z. C., Talukder A. K., Alam M. S., Rahman A. N. M. A. (2014). Different screening tests and milk somatic cell count for the prevalence of subclinical bovine mastitis in Bangladesh. *Tropical Animal Health and Production*.

[8] Sharma N., Pandey V., Sudhan N. A. (2010). Comparison of some indirect screening tests for detection of subclinical mastitis in dairy cows. *Bulgarian Journal of Veterinary Medicine*.

[9] Hegde R., Isloor S., Prabhu K. N. (2013). Incidence of subclinical mastitis and prevalence of major mastitis pathogens in organized farms and unorganized sectors. *Indian Journal of Microbiology*.

[10] Viguier C., Arora S., Gilmartin N., Welbeck K., O'Kennedy R. (2009). Mastitis detection: current trends and future perspectives. *Trends in Biotechnology*.

[11] Lippolis J. D., Reinhardt T. A. (2005). Proteomic survey of bovine neutrophils. *Veterinary Immunology and Immunopathology*.

[12] Van Leeuwen W. B., Melles D. C., Alaidan A. (2005). Host- and tissue-specific pathogenic traits of Staphylococcus aureus. *Journal of Bacteriology*.

[13] Smolenski G., Haines S., Kwan F. Y.-S. (2007). Characterisation of host defence proteins in milk using a proteomic approach. *Journal of Proteome Research*.

[14] Joshi S., Gokhale S. (2006). Status of mastitis as an emerging disease in improved and periurban dairy farms in India. *Annals of the New York Academy of Sciences*.

[15] Madut N. A., Elamin A., Gadir A., Mohamed I., Jalii E. (2009). Host determinants of bovine mastitis in semi-intensive production system of Khartoum state, Sudan. *Journal of Cell and Animal Biology*.

[16] Quinn P. J., Markey B. K., Leonard F. C., Fitzpatrick E. S., Fanning S., Hartigan P. J. (2011). *Veterinary Microbiology and Microbial Disease*.

[17] Dohoo I., Martin W., Stryhn H. (2003). *Veterinary Epidemilogic Research*.

[18] Schukken Y. H., Wilson D. J., Welcome F., Garrison-Tikofsky L., Gonzalez R. N. (2003). Monitoring udder health and milk quality using somatic cell counts. *Veterinary Research*.

[19] Klastrup O., Dodd F. H. Scandinavian recommendations on examination of quarter milk samples.

[20] Markey B., Leonard F. C., Archambault M., Cullinane A., Maguire D. (2013). *Clinical Veterinary Microbiology*.

[21] Iraguha B., Hamudikuwanda H., Mushonga B. (2015). Bovine mastitis prevalence and associated risk factors in dairy cows in Nyagatare District, Rwanda. *Journal of the South African Veterinary Association*.

[22] Mdegela R. H., Ryoba R., Karimuribo E. D. (2009). Prevalence of clinical and subclinical mastitis and quality of milk on smallholder dairy farms in Tanzania. *Journal of the South African Veterinary Association*.

[23] Abebe R., Hatiya H., Abera M., Megersa B., Asmare K. (2016). Bovine mastitis: prevalence, risk factors and isolation of Staphylococcus aureus in dairy herds at Hawassa milk shed, South Ethiopia. *BMC Veterinary Research*.

[24] Plozza K., Lievaart J. J., Potts G., Barkema H. W. (2011). Subclinical mastitis and associated risk factors on dairy farms in New South Wales. *Australian Veterinary Journal*.

[25] Tripura T. K., Sarker S. C., Roy S. K. (2014). Prevalence of subclinical mastitis in lactating cows and efficacy of intramammary infusion therapy. *Bangladesh Journal of Veterinary Medicine*.

[26] Gianneechini R., Concha C., Rivero R., Delucci I. (2002). Occurrence of clinical and sub-clinical mastitis in dairy herds in the west littoral region in Uruguay. *Acta Veterinaria Scandinavica*.

[27] Abrahmsén M., Persson Y., Kanyima B. M., Båge R. (2014). Prevalence of subclinical mastitis in dairy farms in urban and peri-urban areas of Kampala, Uganda. *Tropical Animal Health and Production*.

[28] Mureithi D. K., Njuguna M. N. (2016). Prevalence of subclinical mastitis and associated risk factors in dairy farms in urban and peri-urban areas of Thika Sub County, Kenya. *Livestock Research for Rural Development*.

[29] Östensson K., Lam V., Sjögren N., Wredle E. (2013). Prevalence of subclinical mastitis and isolated udder pathogens in dairy cows in Southern Vietnam. *Tropical Animal Health and Production*.

[30] Kurjogi M. M., Kaliwal B. B. (2014). Epidemiology of bovine mastitis in cows of Dharwad district. *International Scholarly Research Notices*.

[31] Ondiek J. O., Ogore P. S., Shakala E. K., Kaburu G. M. (2013). Prevalence of bovine mastitis, its therapeutics and control in Tatton Agriculture Park, Egerton University, Njoro District of Kenya. *Basic Research Journals of Agricultural Science and Review*.

[32] Ayano A. A., Hiriko F., Simyalew A. M., Yohannes A. (2013). Prevalence of subclinical mastitis in lactating cows in selected commercial dairy farms of Holeta district. *J. Vet. Med. Anim. Heal*.

[33] Hashemi M., Kafi M., Safdarian M. (2011). The prevalence of clinical and subclinical mastitis in dairy cows in the central region of Fars province, south of Iran. *Iranian Journal of Veterinary Research*.

[34] Kayesh M., Talukder M., Anower A. (2014). Prevalence of subclinical mastitis and its association with bacteria and risk factors in lactating cows of Barisal district in Bangladesh. *International Journal of Biological Research*.

[35] Barrett D. J., Healy A. M., Leonard F. C., Doherty M. L. (2005). Prevalence of pathogens causing subclinical mastitis in 15 dairy herds in the Republic of Ireland. *Irish Veterinary Journal*.

[36] Lévesque P. (2004). *Moins de Mammite, Meilleur Lait*.

[37] Sampimon O. C., Riekerink O., Lam T. J. G. M. (2008). Prevalence of subclinical mastitis pathogens and adoption of udder health management practices on Dutch dairy farms: preliminary results. *Mastitis Control: From Science to Practice*.

[38] Thorberg B.-M. (2008). *Coagulase-Negative Staphylococci in Bovine Sub-Clinical Mastitis*.

[39] Hogan J. S., Smith K. L. Occurrence of clinical and subclinical environmental streptococcal mastitis.

[40] Cervinkova D., Vlkova H., Borodacova I. (2013). Prevalence of mastitis pathogens in milk from clinically healthy cows. *Veterinarni Medicina*.

[41] Björk S., Båge R., Kanyima B. M. (2014). Characterization of coagulase negative staphylococci from cases of subclinical mastitis in dairy cattle in Kampala, Uganda. *Irish Veterinary Journal*.

[42] Lim G. H., Leslie K. E., Kelton D. F., Duffield T. F., Timms L. L., Dingwell R. T. (2007). Adherence and efficacy of an external teat sealant to prevent new intramammary infections in the dry period. *Journal of Dairy Science*.

[43] Bagley C. V. Staphylococcus mastitis: Herd control program.

[44] Radostits O. M., Gay C. C., Hinchcliff K. W., Constable P. D. (2007). *Veterinary Medicine: A Textbook of the Diseases of Cattle, Horses, Sheep, Pigs and Goats*.

[45] Wilson D. J., Gonzalez R. N., Das H. H. (1997). Bovine mastitis pathogens in New York and Pennsylvania: prevalence and effects on somatic cell count and milk production. *Journal of Dairy Science*.

[46] Pyörälä S., Taponen S. (2009). Coagulase-negative staphylococci—emerging mastitis pathogens. *Veterinary Microbiology*.

[47] Taponen S. (2008). *Bovine Mastitis Caused by Coagulase-Negative Staphylococci*.

[48] Jones G. M., Bailey T. L., Roberson J. R. (1998). *Staphylococcus aureus Mastitis: Cause, Detection, and Control*.

[49] Radostits O. M., Leslie K. E., Saunders W. B., Leslie K. E., Fetrow J. (1994). *Herd Health: Food Animal Production Medicine*.

[50] Björk S. (2013). *Clinical and Subclinical Mastitis in Dairy Cattle in Kampala, Uganda*.

[51] Parkinson T., Merrall M., Fenwick S. (1999). A case of bovine mastitis caused by Bacillus cereus Continued next page. *New Zealand Veterinary Journal*.

[52] Logan N. A. (1988). Bacillus species of medical and veterinary importance. *Journal of Medical Microbiology*.

[53] Gonzalez R. N. Prototheca, yeast, and bacillus as a cause of mastitis.

[54] Hogan J., Smith K. L. (2003). Coliform mastitis. *Veterinary Research*.

[55] NMC (2010). *A Practical Look at Environmental Audits*.

[56] Kivaria F. M., Noordhuizen J. P. T. M., Nielen M. (2007). Interpretation of California mastitis test scores using Staphylococcus aureus culture results for screening of subclinical mastitis in low yielding smallholder dairy cows in the Dar es Salaam region of Tanzania. *Preventive Veterinary Medicine*.

[57] Bradley A. J., Green M. J. (2001). Aetiology of clinical mastitis in six Somerset dairy herds. *Veterinary Record*.

[58] Kremer W. D., Noordhuizen-Stassen E. N., Lohuis J. A. (1990). Host defence and bovine coliform mastitis. Host defence mechanisms and characteristics of coliform bacteria in coliform mastitis in bovine: a review. *Veterinary Quarterly*.

